# Identification of additional mitochondrial DNA mutations in canine mast cell tumours

**DOI:** 10.1186/s13028-016-0210-y

**Published:** 2016-05-04

**Authors:** Anna Śmiech, Brygida Ślaska, Magdalena Surdyka, Ludmiła Grzybowska-Szatkowska, Wojciech Łopuszyński, Dorota Różańska

**Affiliations:** 1 Department of Pathological Anatomy, Faculty of Veterinary Medicine, University of Life Sciences in Lublin, Głęboka 30 St., 20-612 Lublin, Poland; 2 Department of Biological Bases of Animal Production, Faculty of Animal Breeding and Biology, University of Life Sciences in Lublin, Akademicka 13 St., 20-950 Lublin, Poland; 3 Department of Oncology, Medical University of Lublin, Jaczewskiego 7 St., 20-090 Lublin, Poland; 4 Department and Clinic of Animal Surgery, Faculty of Veterinary Medicine, University of Life Sciences in Lublin, Akademicka 13 St., 20-950 Lublin, Poland

**Keywords:** Tumour, Dog, D-loop, mtDNA, Mutations

## Abstract

**Background:**

Research has revealed the presence of somatic mutations in mitochondrial DNA (mtDNA) of certain types of tumours. As this has not been studied for canine mast cell tumours, the aim of this study was to identify mutations in the hypervariable region of mtDNA in mast cell tumours in dogs and determine their association with the process of neoplastic transformation.

**Results:**

Samples from 17 dogs with histopathologically confirmed mast cell tumours were analysed. The samples consisted of tumour tissues (n = 17), normal tissues (n = 17), and blood (n = 17). Amplicons of the displacement loop (D-loop) were sequenced and the obtained nucleotide sequences were subjected to bioinformatics analyses. Somatic mutations were detected in seven positions of the D-loop nucleotide sequences in 47 % of the dogs, while polymorphisms were identified in 94 % of the dogs. Most of these changes were homoplasmic, while heteroplasmy was detected in two individuals. Six new haplotypes were established as being characteristic for canine mast cell tumours. There was no association between the presence of the mutations and sex, haplotype, or malignancy grade assessed in 3 and 2-grade scales.

**Conclusions:**

Differences in the frequency of somatic mutations imply their direct association with the neoplastic transformation. However, their functional consequences and clinical significance are not clear. The mutations may be used for diagnosis and prognosis of canine mast cell tumours in the future.

## Findings

Mast cell tumours account for 7–21 % of all diagnosed skin neoplasms in dogs [1]. The aetiology of mast cell tumour development is not fully understood but recent research of human tumours has revealed the presence of somatic mutations in mitochondrial DNA (mtDNA), in particular in the D-loop region, which controls replication and transcription of mtDNA [2, 3].

Somatic cells contain hundreds to several thousand mitochondria, each containing 1–10 gene copies of mtDNA, which often undergoes spontaneous mutations due to their lack of protective activity of histones, the globular, coiled structure of mtDNA and a high level of mitochondrial reactive oxygen species (ROS). When a cell or tissue contains both mutated and normal wild-type mtDNA, the condition is known as heteroplasmy, while homoplasmy refers to the presence of only one type of mtDNA (mutated or normal mtDNA). Most changes within the mtDNA nucleotide sequences of neoplastic cells are homoplastic [3].

Investigations of the association between mtDNA somatic mutations and neoplastic transformation have primarily been conducted in humans [2–4] where somatic mtDNA mutations have been found in malignant tumours such as prostate and breast cancer. Such mutations are considered to trigger the neoplastic transformation through shifts of cell energy resources, an increase in the mitochondrial oxidative stress, and modulation of apoptosis [3]. Only a few studies have been carried out on canine tumours [5–9]. Bertagnolli et al. [5] investigated mixed canine mammary tumours and performed an analysis of a fragment of the D-loop of mtDNA from epithelial and mesenchymal tumour components. They identified nucleotide changes that could serve as molecular markers for establishing the origin of neoplastic cells. An association between a nuclear and/or mtDNA mutation and malignant transformation has been observed for canine tumours in other studies [6–9]. Slaska et al. [7, 8] observed somatic mutations in the D-loop region of mtDNA in 62 % of examined epithelial tumours and in 25 % of mesenchymal tumours. In another study, no mutations in mesenchymal tumours were been found, whereas 50 % of epithelial tumours exhibited somatic mutations. Blood and tumour heteroplasmy in the D-loop region has been diagnosed in dogs with squamous cell carcinoma, comedo carcinoma of the mammary gland, and sweat gland carcinoma [7, 8].

Besides the D-loop, three mtDNA genes, i.e. the NADH dehydrogenase subunit 1 (*ND1*), cytochrome c oxidase subunit I (*COI*), and cytochrome b (*CYTB*) have been analysed in different canine tumour types. The results showed that polymorphisms in these genes influenced the function of proteins and thus their potential role in carcinogenesis [9].

Presence of mtDNA defects in canine mast cell tumours has not been reported. The aim of this study was therefore to identify mutations in the hypervariable region of mtDNA in canine mast cell tumours and to assess their association with the process of neoplastic transformation.

Complete sets of tumour tissues, normal skin tissues and blood (n = 17) were obtained from 17 dogs with mast cell tumours that had undergone surgery. Normal and tumour tissues were sampled for histopathology and were fixed in buffered formalin, pH = 7.2, processed routinely, embedded in paraffin wax, sectioned at 4 µm and stained with haematoxylin-eosin and toluidine blue. Microscopic classification was performed in accordance with the WHO histological classification [10]. The degree of malignancy was assessed using both a 3-grade scale [11] and a 2-grade scale [12]. The dogs were divided into three groups according to age: I (up to 5 years), II (from 6 to 9 years), and III (over 10 year-old).

DNA was extracted from tissues and blood with the DNeasy Blood & Tissue Kit (Qiagen, Hilden, Germany). DNA samples were assessed quantitatively and qualitatively by electrophoretic separation in agarose gel and spectrophotometrically by measurements of sample absorbance in a BioPhotometer spectrophotometer (Eppendorf, Hamburg, Germany). Amplification of the D-loop was performed, using a polymerase chain reaction (PCR) technique in a T100 Thermal Cycler (Bio-Rad, Wroclaw, Poland). Primer sequences used in the analysis, encompassing a mtDNA fragment between nucleotide 15746–16107 (LF 5′- CATACTAACGTGGGGGTTAC; HR 5′- CCATTGACTGAATAGCACCTTG), were based on already published data [13]. The annealing temperature (Ta-60.8 °C) and amplification conditions were established experimentally. Amplification products were visualized on 2 % agarose gel. Amplicons were sequenced using a BigDye Terminator Cycle Sequencing kit (Applied Biosystem, Foster City, CA, USA) in GeneAmp PCR system 9700 (Applied Biosystem). The samples were subsequently purified on CentriSep columns according to the manufacturer’s protocol or precipitated with ethanol and sodium acetate according to the protocol of the BigDye kit manufacturer. Extension products were separated on an ABI 377 automated sequencer (Applied Biosystem).

The obtained nucleotide sequences were subjected to bioinformatics analyses in order to determine mutation and polymorphic sites within the mtDNA fragment of each sample (DNA Baser Sequence Assembler v 3.2). The D-loop nucleotide sequences were compared to the reference sequence [GenBank: U96639] [14, 15] and described as the polymorphisms. The hypervariable region I (HVI) dog haplotypes were established according to Savolainen et al. [16], Pereira et al. [17], and Imes et al. [18].

The probability of the presence of a mutation in each locus in relation to the age, sex, haplotype, and malignancy grade was estimated using the method of least-squares means (lsm) ± standard error (se). The correlation between the data was analysed using the SAS 9.4 procedure PROC GLM (SAS Institute, Cary, NC, USA). Correlations with P ≤ 0.05 were considered significant.

The study was approved by the II Local Ethical Commission for Animal Experiments in Lublin, Poland (Resolution number 6/2013).

**Fig. 1:**
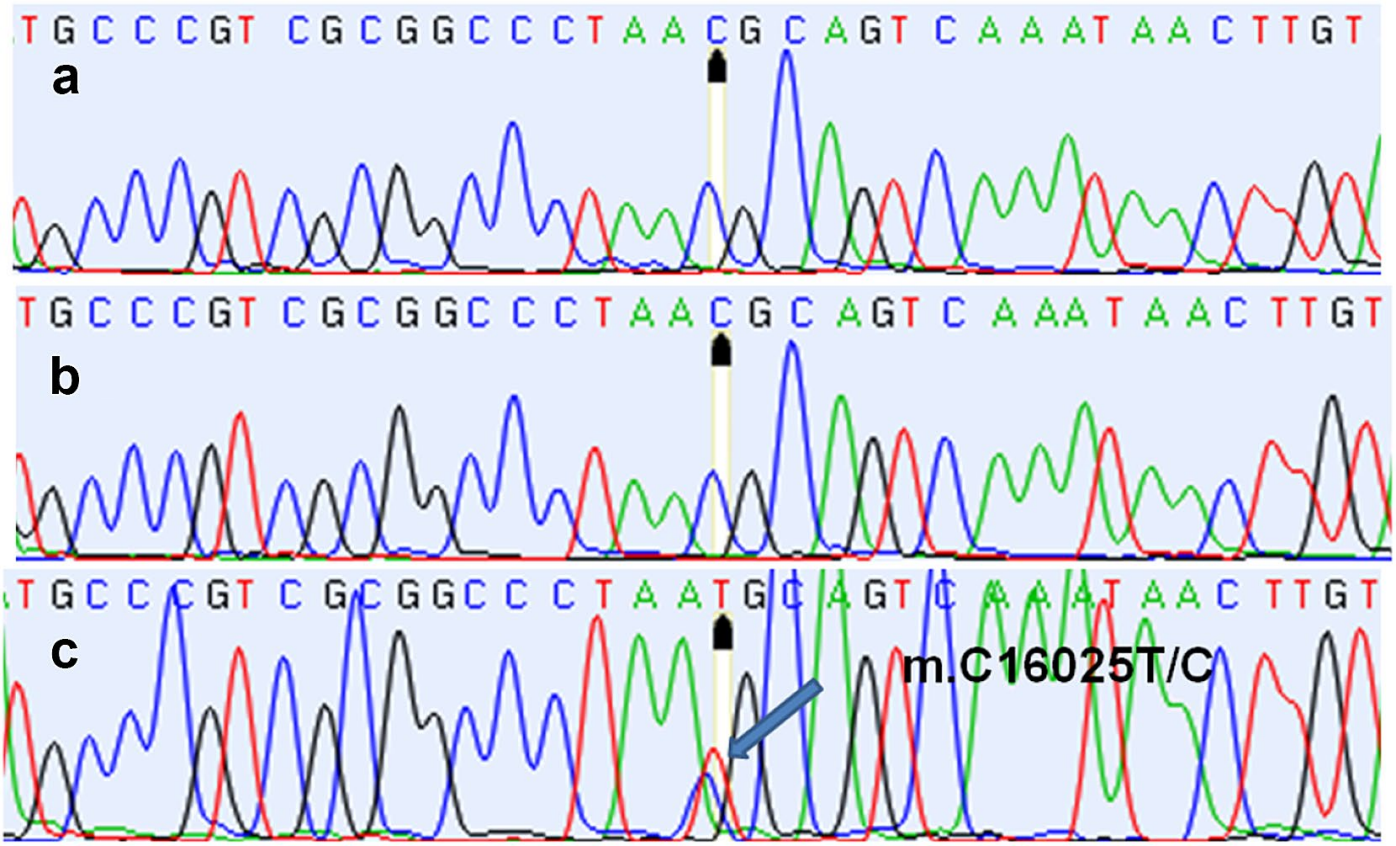
Cancer heteroplasmy in position m.16025 (**a** normal tissue, **b** blood, **c** tumour tissue)

**Fig. 2:**
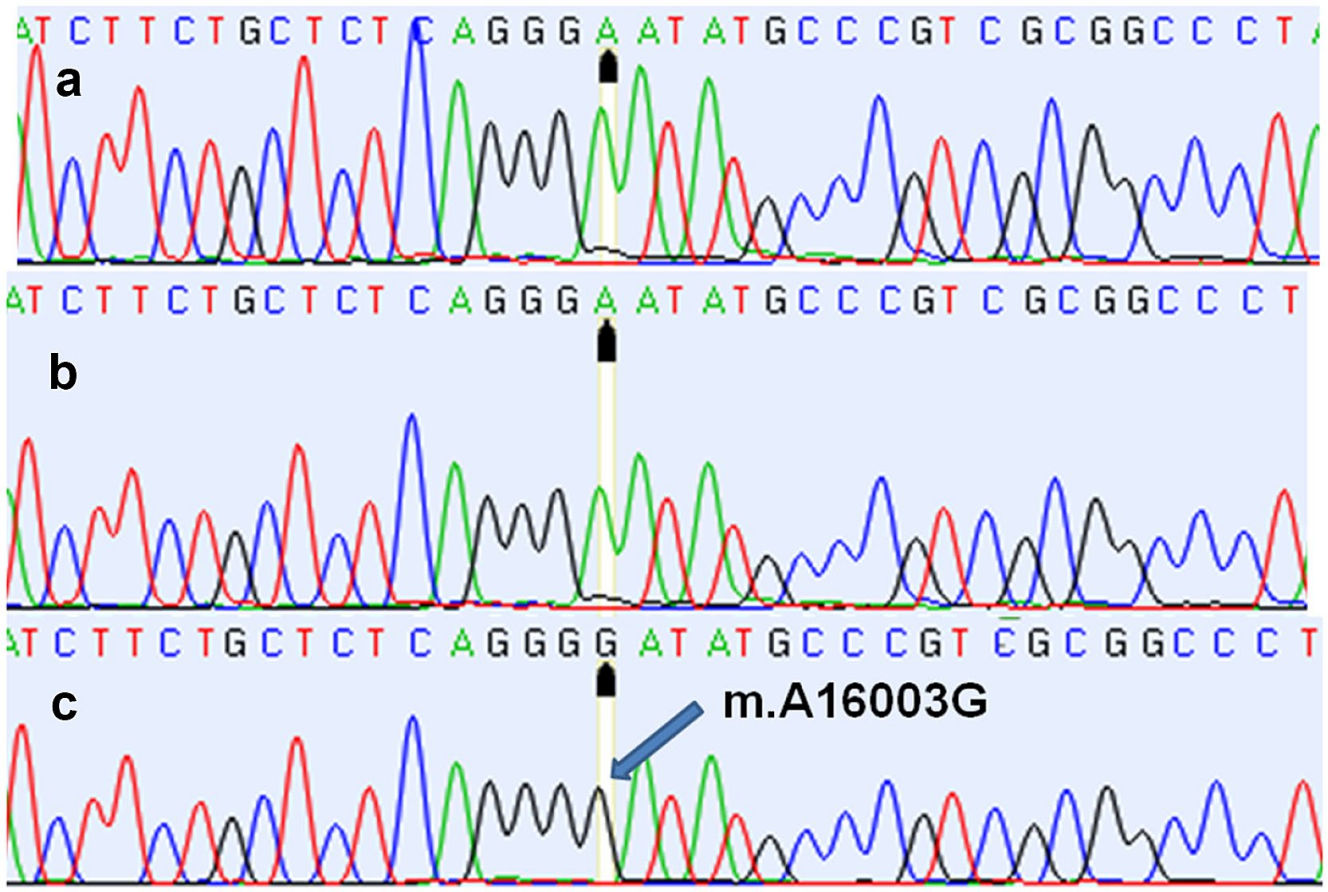
Mutation in tumour tissue in position m.16003 (**a** normal tissue, **b** blood, **c** tumour tissue)

Data on sex, breed, age and malignancy grade of the dogs are presented in Table 1. Mutations and/or polymorphisms were observed in eight positions of the D-loop nucleotide sequences in all cases (n = 17) of mast cell tumours (Tables 2, 3). Somatic mutations in seven positions of the D-loop nucleotide sequences were detected in 47 % of the dogs (Table 2). All changes were substitution mutations, i.e. exchange of one nucleotide base with another. These mutations were detected in the tumour tissue and blood, but not in the normal tissues. The majority of the changes were homoplasmic. In one dog, heteroplasmy was detected in the blood and tumour in position m.C15815C/T (Table 2); in another dog, heteroplasmic changes were found only in the tumour in positions m.C15912T/C and m.C16025T/C (Fig. 1). Somatic mutations in tumour tissues in positions m.16003 (Fig. 2) and m.15912 mtDNA exhibited the highest frequency, in approximately 35 and 24 % of the cases, respectively. These mutations were also detected in the blood of one dog.

**Table 1:** Data on dogs with mast cell tumours included in the study

Dog number	HVI dog haplotype	Sex	Breed	Age (years)	Grading according to Patnaik et al. [11], Kiupel et al. [12]
1	B1	F	Dachshund	9	Grade III/high-grade
2	A18	F	Bavarian Mountain Hound	9	Grade III/high-grade
3	A26	F	St. Bernard	6	Grade II/high-grade
4	B11	M	French Bulldog	10	Grade II/low-grade
5	B1	F	Crossbreed	6	Grade I/low-grade
6	A17	M	Boxer	8	Grade I/low-grade
7	B1	F	Schnauzer	8	Grade II/low-grade
8	A18	F	Boxer	8	Grade II/low-grade
9	A18	F	Golden Retriever	4	Grade I/low-grade
10	A18	F	Central Asian	4	Grade II/high-grade
11	A18	F	Crossbreed	8	Grade II/low-grade
12	A17	M	Labrador	10	Grade II/low-grade
13	A19	F	Crossbreed	11	Grade II/low-grade
14	A26	F	Labrador	3,5	Grade II/low-grade
15	A18	F	Bernese Mountain Dog	8	Grade II/high-grade
16	A18	F	Crossbreed	15	Grade II/low-grade
17	A18	F	Bernese Mountain Dog	9	Grade III/high-grade

**Table 2:** Somatic mutations in the D-loop sequence in normal tissue, blood and tumour tissue in cases of canine mast cell tumours

Dog number	Sequences in normal tissue	Sequences in blood	Sequences in tumour tissue	HVI haplotype (normal tissue → tumour tissue)
1	m.15815C	m.15815C/T	m.15815C/T	B1 → B1/B37
2	m.15800T	m.15800T	m.15800C	A18 → B37
	m.15912C	m.15912C	m.15912T	
	m.15955C	m.15955C	m.15955T	
	m.16003A	m.16003A	m.16003G	
3	m.15912C	m.15912C	m.15912T/C	A26 → A101*/102*
	m.16003A	m.16003A	m.16003G	
	m.16025C	m.16025C	m.16025T/C	
4	m.15800 T	m.15800 T	m.15800 C	B11* → A172*
5	m.15800C	m.15800T	m.15800T	B1 → A17
	m.15815C	m.15815T	m.15815T	
	m.15912T	m.15912C	m.15912C	
	m.16003G	m.16003A	m.16003A	
6	m.15795C	m.15795C	m.15795T	A17 → A173*
7	m.16003G	m.16003G	m.16003A	B1 → B12*
13	m.16003A	m.16003A	m.16003G	A19 → A191*

Somatic mutations in the D-loop sequence were present in 8 out of 17 dogs

* Recently reported haplotypes [16–18]

**Table 3:** Differences in the mitochondrial D-loop region between the reference sequence and normal tissue, blood and tumour tissue sequences in cases of canine mast cell tumours

Dog number	Reference sequence	Sequence in normal cells	Sequence in blood	Sequence in tumour cells
1	m.15800T	m.15800C	m.15800C	m.15800C
1–12, 14–17	m.15814C	m.15814T	m.15814T	m.15814T
7	m.15815T	m.15815C	m.15815C	m.15815C
1, 7	m.15912C	m.15912T	m.15912T	m.15912T
1, 4–7, 12	m.15955C	m.15955T	m.15955T	m.15955T
1	m.16003A	m.16003G	m.16003G	m.16003G
14	m.16025T	m.16025C	m.16025C	m.16025C

Differences were not present in one of the studied dogs (no. 13)

Recently reported tumour haplotypes (except B11) were present in tumour tissue but not in normal tissue or blood (Table 2). The HVI dog haplotype A18, accounting for 41.7 % in normal tissue and blood, was the most prevalent haplotype in each type of the analysed tissues. In tumour tissue, it was present in 41.2 % of the tumours. The mtDNA haplotype was changed in all dogs with detected mutations; hence, six not previously reported haplotypes of mast cell tumours were detected. Compared to the reference canine sequence, polymorphisms (m.T15800C, m.C15814T, m.T15815C, m.C15912T, m.C15955T, m.A16003G, m.T16025C) were found in seven positions of the D-loop sequences in 16 of 17 dogs (94 %) with mast cell tumours (Table 3). In most dogs (94 %) polymorphism was detected in position m.C15814T, while polymorphism at D-loop position m.C15955T was found in 35 % of the examined dogs (Table 3).

Mutations in loci m.15912 and m.16003 were only detected in dogs aged six year or older (age groups II and III). The risk of mutation in locus m.16003 was significantly higher in the oldest dogs (age group III) compared to the youngest dogs (P = 0.047). However, since the group sizes were limited, these data should be interpreted with care. Statistical analyses did not reveal an association between presence of mutations and sex, haplotype, or the tumour malignancy grades (Tables 1 and 2).

Analysis of the D-loop sequences revealed a relatively high genetic variability. As the mutations occurred independently of the mast cell tumour malignancy grades, this indicates that the presence of mutations in the D-loop and the malignancy grade was not associated. Mutations in the mtDNA sequences and sex or haplotype were not associated as also observed for other types of canine tumours [7–9]. Some of the observed mutations (except m.16003 and m.16025) have been recognized in other types of canine tumours previously [5]. These were mostly homoplasmic substitution mutations although single heteroplasmy mutations were found as well.

Presence of mutations in the D-loop sequences in tumour tissue and/or blood of dogs with mast cell tumours indicates that they may be involved in the process of neoplastic transformation. Further studies on the significance of mtDNA D-loop mutations and their association with neoplastic transformation, biological behaviour, and histologic grade of canine mast cell tumour are however required to determine their significance.
